# Supplementary material for: Improved in vivo gene knockout with high specificity using multiplexed Cas12a sgRNAs
Source: Nat Commun. 2026 Jan 15;17:877. doi: 10.1038/s41467-026-68434-z (PMC12827956; doi:10.1038/s41467-026-68434-z)
Supplement: Supplementary file 1 — Supplementary Information [file 41467_2026_68434_MOESM1_ESM.pdf]

Supplementary Information for "Improved *in vivo* gene knockout with high specificity using multiplexed Cas12a sgRNAs"

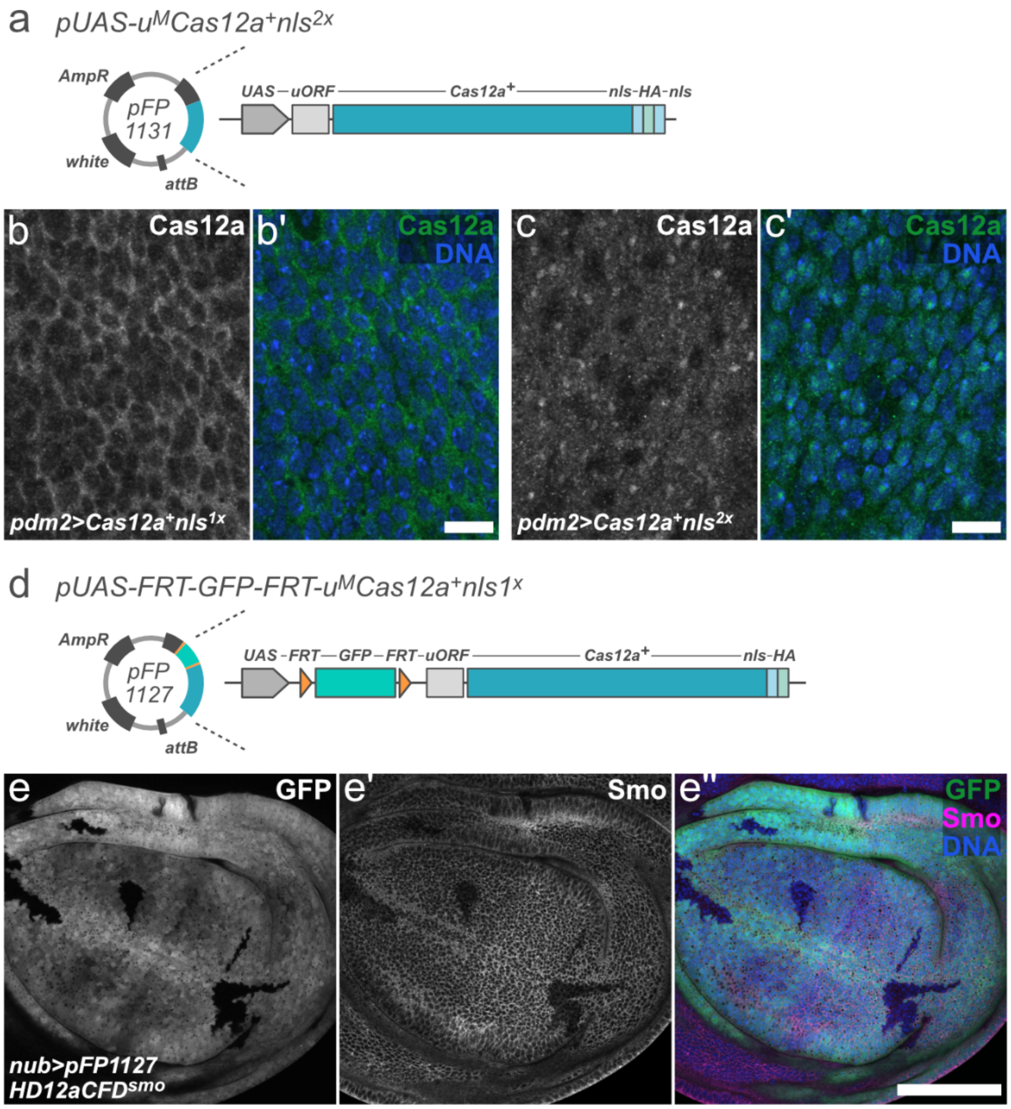

Supplementary Figure 1: Transgenic tools for conditional Cas12a^{+} mutagenesis. (**a**) Schematic representation of the *pUAS-u^{M}Cas12a^{+}nls^{2x}* construct (*pFP1131*) containing UAS-driven Cas12a with two nuclear localization signals (nls). The upstream open reading frame (uORF) attenuates Cas12a^{+} expression[1] and additional NLSs have been shown to increase editing efficiency in mammalian cell[3,4]. (**b - b'**, **c - c'**) Immunofluorescence showing Cas12a expression (grayscale and green) with DNA counterstain (blue) in wing discs. Scale bars: 10 µm. A previously described Cas12a^{+} construct (25) with a single NLS localizes predominantly to the cytoplasm (b). Cas12a^{+} encoded on *pFP1131* localizes more efficiently to the nucleus (c). (**d**) Schematic of the *pUAS-FRT-GFP-FRT-u^{M}Cas12a^{+}nls^{1x}* construct (pFP1127) featuring a FRT-flanked GFP transgene upstream of Cas12a for inducible expression through FLP-mediated cassette excision. (**e - e''**) Cas12a^{+} editing with spatial and temporal control.

Immunofluorescence showing GFP (grayscale/green), Smoothened (Smo) (grayscale/magenta), and DNA (blue) in *hs-FLP nub-Gal4 UAS-pUAS-FRT-GFP-FRT-u*^{M}*Cas12a*$^{+}$*nls*^{1x} *HD12aCFD*^{smo} wing imaginal discs approximately 60 h after a limited heat shock. Smo expression is selectively and efficiently lost in cells that excised the FRT-GFP cassette. Scale bar: 50 µm.

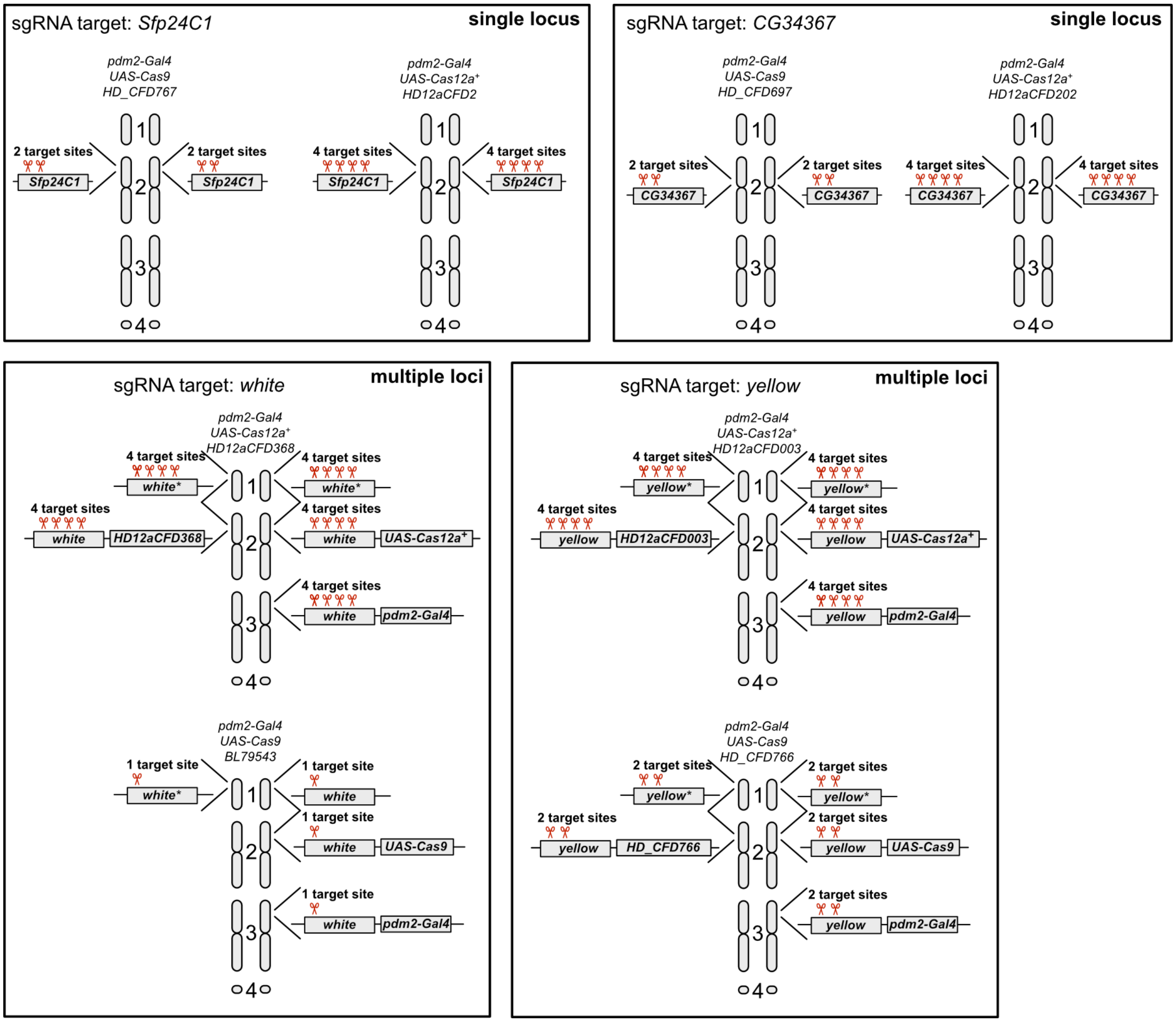

Supplementary Figure 2: Genotypes and sgRNA target sites of strains used to test for DSB induced toxicity. Schematic of the genotypes of each strain used in Fig. 3. sgRNA target sites are indicated by red scissors. Target genes *Sfp24C1* and *CG34367* are both present in a single copy on chromosome arm 2L. They were chosen because they are assumed to be non-essential for cells of the wing epithelium and LOH experiments showed that these sgRNa lines are highly active. The genes *white*

and *yellow* are used to mark transgenes or the landing sites they are inserted in and are therefore present on multiple chromosomes. Note that to target *white* with Cas9 we used a line encoding a single sgRNA (BL79543, a *white* targeting HD_CFD line does not exist). BL79543 is marked by *vermillion*, not *white*. Despite the low number of target sites this line still mediates increased cell death in the wing imaginal disc (Fig. 3b).

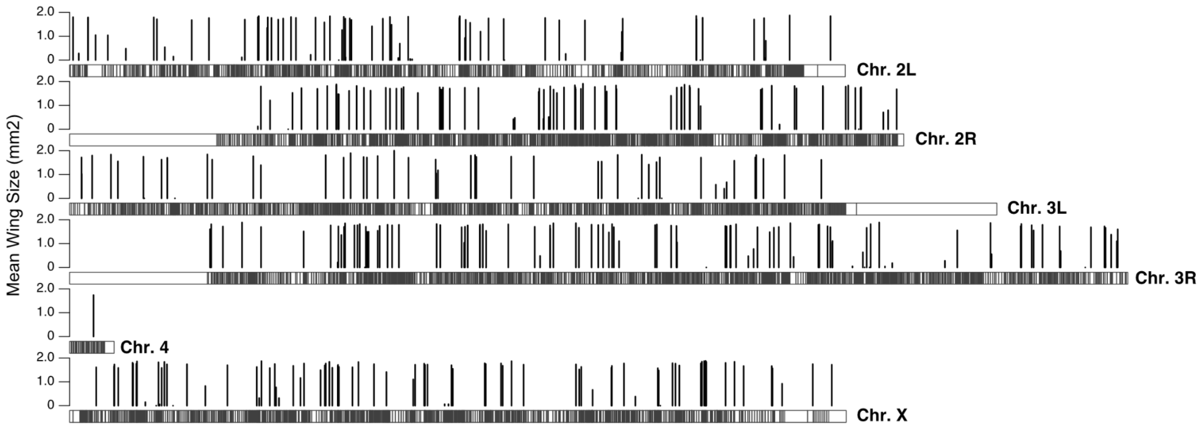

Supplementary Figure 3: Wing size phenotypes from Cas12a^{+}-mediated mutagenesis show no correlation with chromosomal position of target genes. Mean wing size measurements (mm^2) are plotted at the genomic location of each targeted gene, with bars positioned relative to the chromosome ideograms shown below. Each vertical bar represents the mean wing area from approximately 10 flies expressing Cas12a^{+} under *pdm2-Gal4*, combined with a HD12aCFD sgRNA array targeting the respective genomic locus. Bars are positioned according to target gene coordinates using the karyoploteR R package. Note that centromeres are positioned toward the right for chromosomes 2L, 3L, and 4, and toward the left for chromosomes 2R, 3R, and X. Genes whose disruption produces reduced wing size are distributed randomly across all chromosomes with no apparent clustering, indicating that the observed phenotypes result from specific gene targeting rather than chromosomal position effects. Individual replicate data for each line are provided in Supplementary Fig. 9.

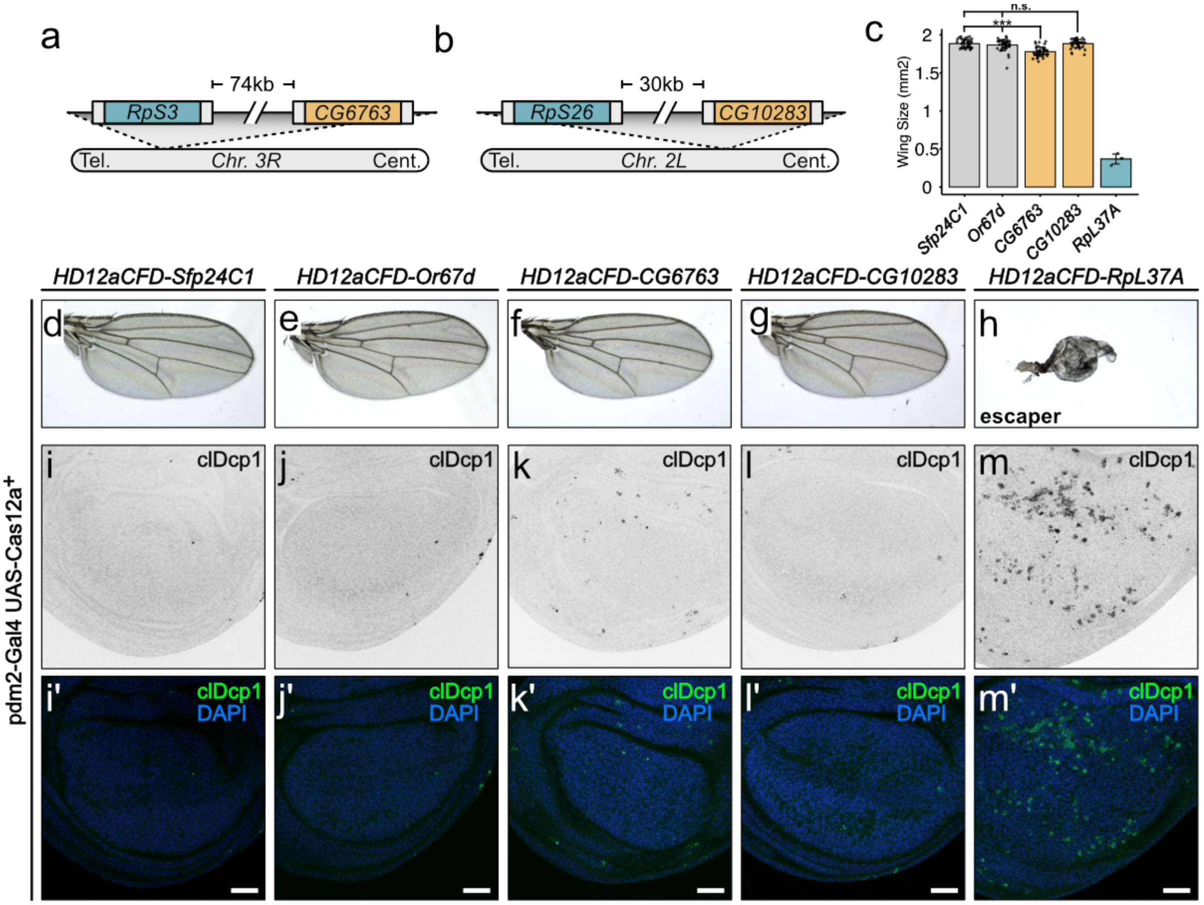

Supplementary Figure 4: Effect of targeting genes in proximity to haploinsufficient Minute genes with Cas12a$^+$ and HD12aCFD arrays. (**a** - **b**) Schematics representing the location of the two target genes in (f) and (g) relative to known Minute genes. (**c**) Wing size of flies expressing Cas12a$^+$ under *pdm2-Gal4* and a HD12aCFD sgRNA array targeting the indicated genes. *Sfp24C1* and *Or67d* play no role in wing morphogenesis and are considered negative controls. *RpL37L* is a Minute gene and gene targeting resulted in lethality, but two flies survived and their wings were measured. Targeting *CG6763* resulted in a mild, but significant reduction of wing size compared to both controls, while targeting *CG10283* had no effect (bars = mean; error bars = standard deviation; points = individual measurements; n.s. = not significant; *** = p<0.001; two-tailed pairwise Welch's T-test with Bonferroni correction). (**d** - **h**) Representative images of wings of the indicated genotypes. The degenerated wing in (h) is from one of only two surviving flies (from >50 animals). Morphology of wings from genotypes depicted in d - g was largely normal, with occasional notches of the wing margin observed in *pdm2-Gal4 UAS-Cas12a$^+$ HD12aCFD-CG6763* flies. (**i** - **m**) Apoptotic cells in the wing disc epithelium. Apoptosis was detected by presence of cleaved Dcp-1 caspase and strongly elevated in tissues with gene targeting of *RpL37A*, slightly elevated when targeting *CG6763* and comparable in the other genotypes. Scale bar = 50 um. Together, these data are consistent with low level proximity effects when targeting *CG6763* (although observed phenotypes could also indicate a role in cell fitness of *CG6763* itself), while no such effects were detected when targeting *CG10283*.

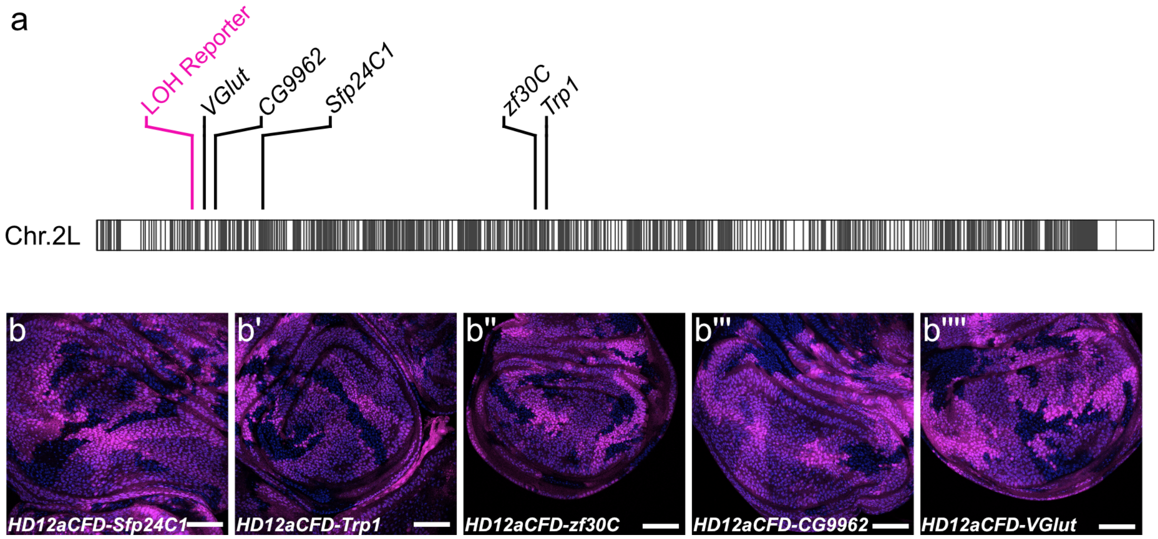

Supplementary Figure 5: Loss of heterozygosity is copy-number neutral in *Drosophila* wing discs. (**a**) Ideogram of chromosome arm 2L with telomere to the left and centromere to the right. Location of the LOH reporter and the five target genes are indicated above. (**b - b''''**) Representative images of wing imaginal discs expressing Cas12a^{+}, a sgRNA array targeting the indicated gene, and the heterozygous fluorescent reporter (shown in magenta). Note that loss of heterozygosity results in cells with high levels of fluorescence and loss of fluorescence in roughly equal ratios. Quantifications are presented in Fig. 5c. Scale bar = 50 µm.

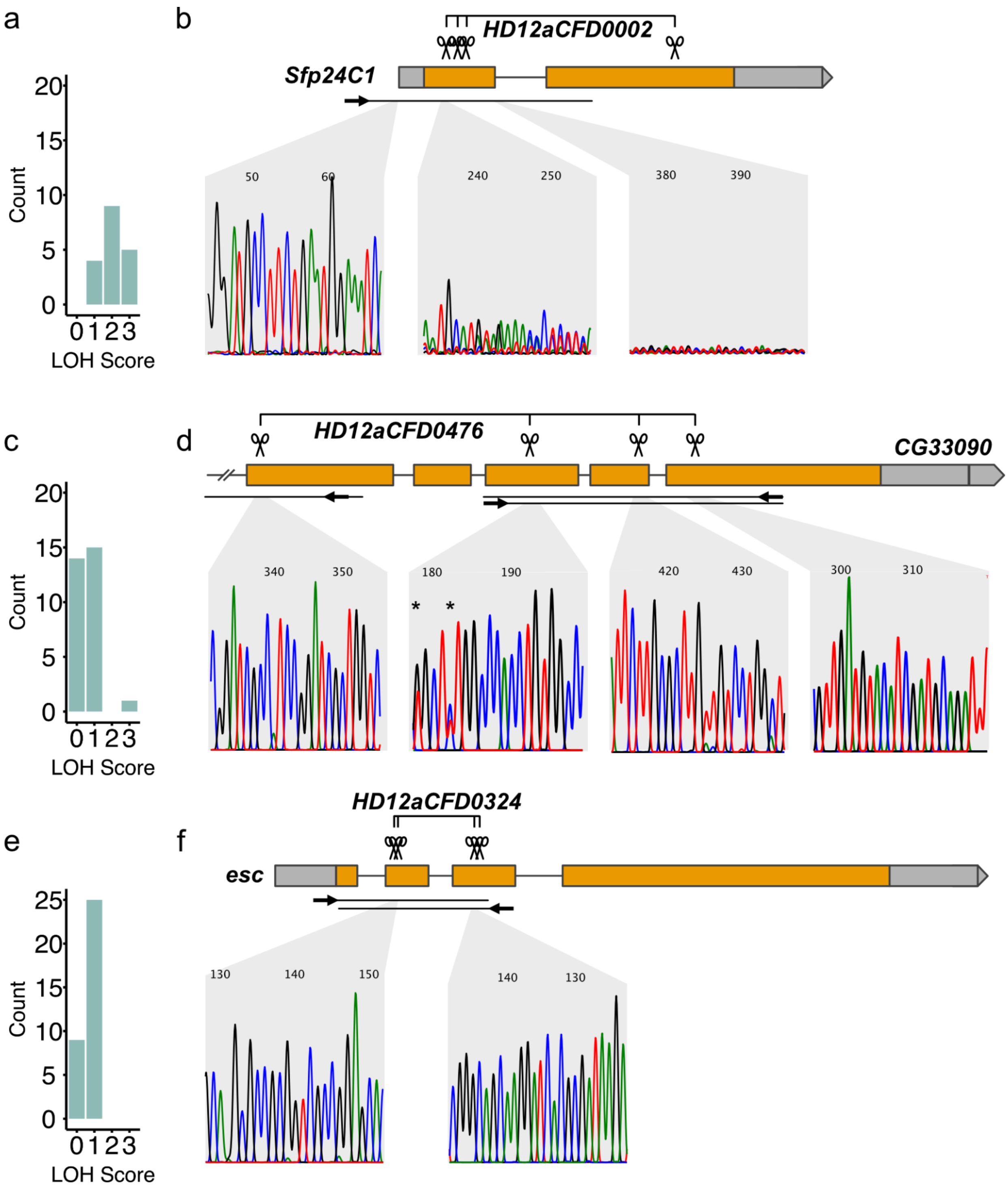

Supplementary Figure 6: LOH screening is sensitive enough to detect nuclease activity mediating low on-target mutagenesis. Correlation between detection of LOH and mutations at the sgRNA target sites detected by Sanger sequencing. (**a**) Line HD12aCFD0002 results in robust LOH in wing imaginal discs, with all samples scoring as either weak (LOH Score = 1), medium (LOH Score =2) or strong (LOH Score = 3) for LOH. Screening was performed as in Fig. 5f-j in heterozygous *act-Cas12a*$^{+}$ *GMR11F02-*

Gal4 UAS-GFP HD12aCFD sgRNA array animals. (**b**) Genomic context and sequencing traces for the target locus of HD12aCFD0002, which results in strong sequence diversification at the sgRNA target sites. Gene models (not drawn to scale) show exons (orange boxes), untranslated regions (gray boxes), and sgRNA target sites (scissors symbols), with Sanger sequencing chromatograms of the region indicated by the grey triangles shown below. (**c** - **f**) LOH Scores and sequencing chromatograms for lines HD12aCFD0476 and HD12aCFD0324. Both lines result in weak LOH, with detection in some, but not all samples (**c**, **e**). Sanger sequencing reveals minimal to no detectable sequence diversification at the sgRNA target sites (**d**, **f**). Asterisks in panel (d) indicate sequence polymorphisms that are also found in a control genotype (note that this also indicates that both alleles are PCR amplified, excluding the presence of a large deletion at the target locus). Genomic DNA was extracted from single *act-Cas12a*$^{+}$ *GMR11F02-Gal4 UAS-GFP HD12aCFD* sgRNA array animals and the target locus was amplified by PCR and amplicons were analyzed by Sanger sequencing. *act-Cas12a+* mediates mutagenesis independently in different cells of the same animal, creating complex genetic mosaics and resulting in an overlay of different sequences in Sanger sequencing chromatograms from amplicons of edited loci. Together, this suggests that LOH screening has similar sensitivity to detect CRISPR activity than Sanger sequencing and might indicate that CRISPR induced DNA breaks can in some cases result in mitotic recombination without concomitant mutations at the break point.

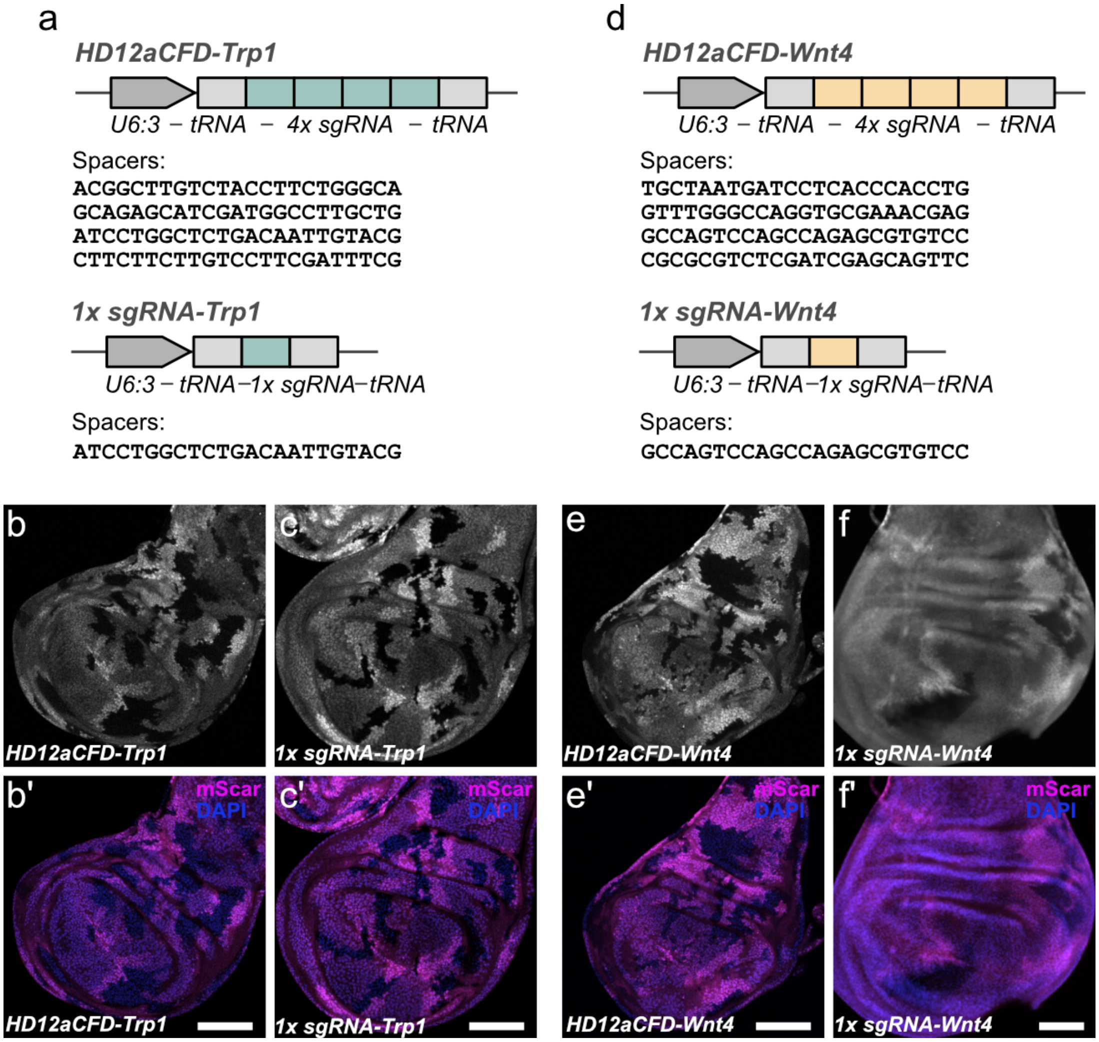

Supplementary Figure 7: Single sgRNAs induce robust loss of heterozygosity. Single sgRNAs are sufficient to induce LOH in combination with Cas12a⁺. (**a**) Schematic of the constructs used in (b) and (c). The HD12aCFD sgRNA array encodes four sgRNAs targeting *Trp1*. For the 1x sgRNA construct one of the sgRNAs has been cloned into a vector with otherwise identical sequence to test the potency of single sgRNAs for LOH induction. (**b**-**b'**) Representative image of a wing imaginal disc expressing Cas12a⁺, the HD12aCFD-Trp1 array and a heterozygous mScarlet reporter gene. LOH results in cells with increased (gain of reporter) or decreased (loss of reporter) fluorescence. (**c**-**c'**) A similar phenotype than in (b) is observed when Cas12a⁺ is combined with just a single sgRNA. (**d**) Schematic of the constructs used for the experiments presented in (e) and (f). (**e**-**e'**) Targeting *Wnt4* with Cas12a⁺ and a HD12aCFD sgRNA array results in frequent LOH. (**f**-**f'**) Similar, albeit less frequent LOH is observed when *Wnt4* is targeted by only a single sgRNA. Together this demonstrates that activity of single sgRNAs can be reliably detected in wing imaginal discs. Nuclei are stained in blue (DAPI) and the LOH reporter is shown in magenta (mScar). Scale bars = 50 um. Images in (b),(c), and (e) a single confocal sections, while for technical reasons (microscope undergoing repair) the image in (f) was acquired with a widefield fluorescent microscope.

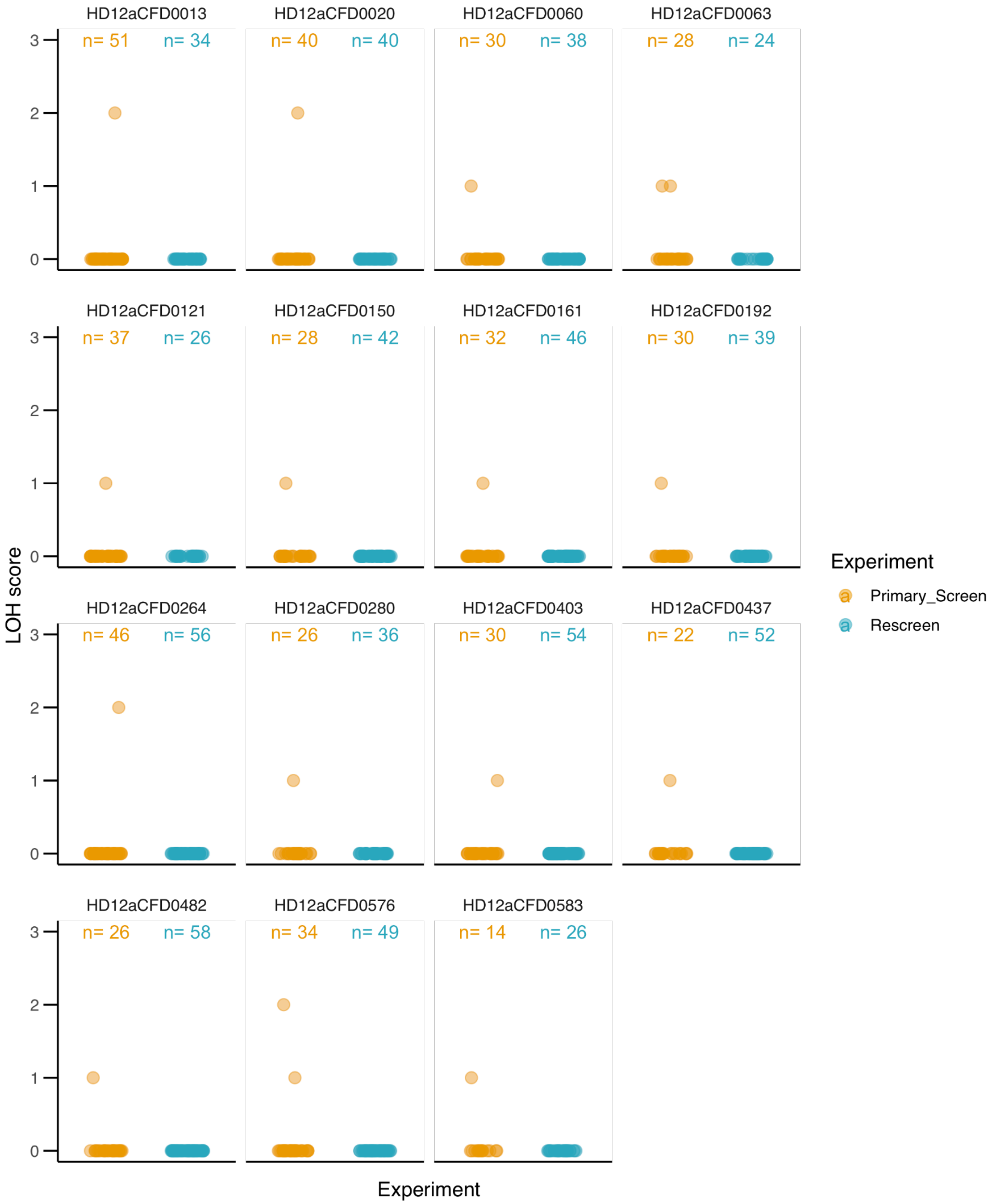

Supplementary Figure 8: Sporadic LOH is not reproducible. HD12aCFD lines that resulted in sporadic LOH in the primary screen on chromosome arm 2L were rescreened using the same conditions, but with increased sample sizes. No LOH was detected in the rescreen, indicating that the initial signal does not represent reproducible CRISPR off-targets, but is more likely to reflect either stochastic LOH or experimental error during the high-throughput screen.

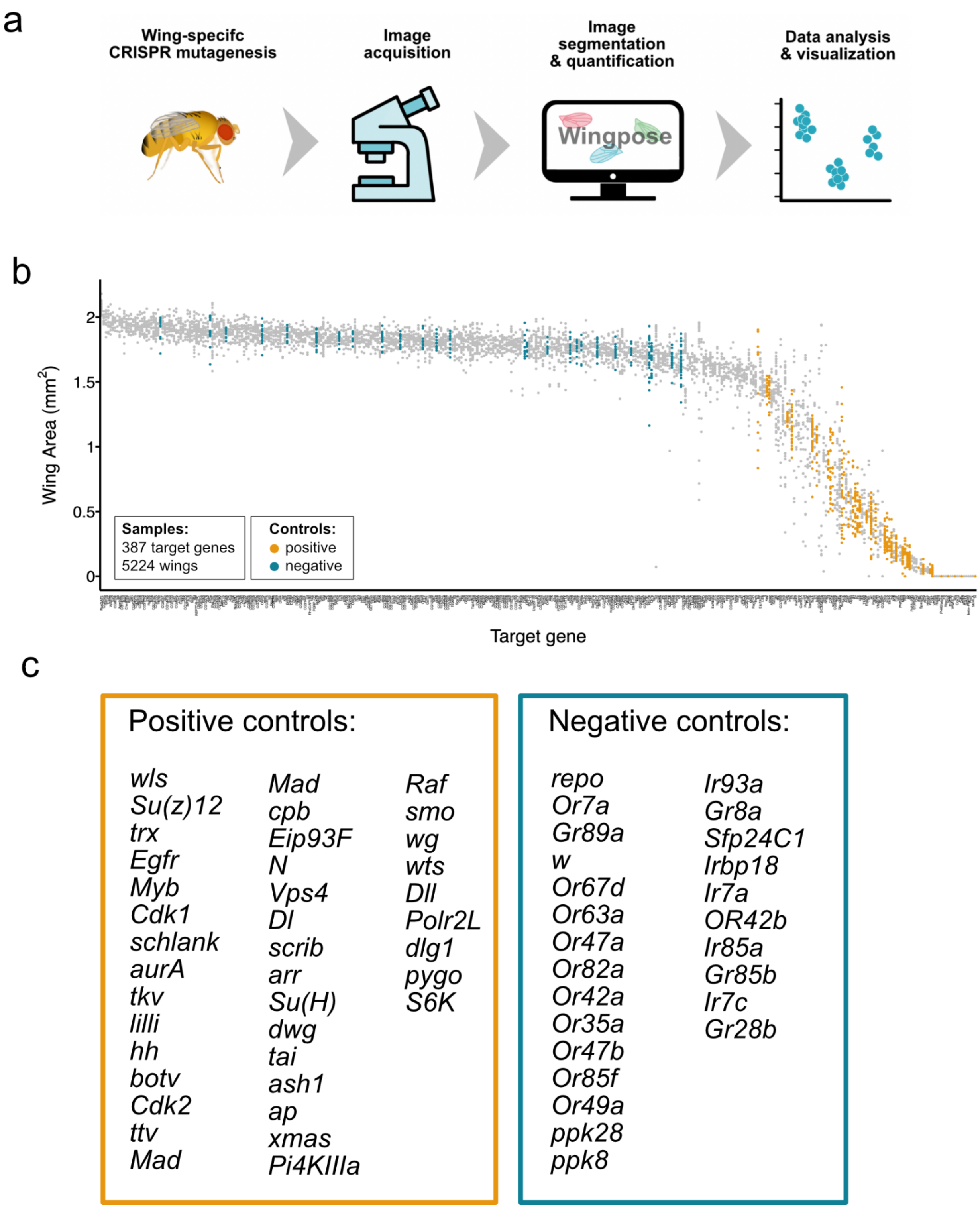

Supplementary Figure 9: Tissue-specific CRISPR mutagenesis in wings with Cas12a$^+$ and

HD12aCFD sgRNA arrays. (**a**) Schematic of the experimental workflow. Tissue-specific CRISPR mutagenesis in wing precursor cells is induced by *pdm2-Gal4 UAS-Cas12a+* and HD12aCFD sgRNA arrays. Wings are mounted, imaged and segmented with a custom trained Cellpose model (Supplementary Data 4). Wing size is then measured in Fiji and analysed. (**b**) Size of individual wings from animals expressing Cas12a+ and HD12aCFD sgRNA arrays targeting 387 genes. Each dot presents an individual wing (with typically between 10 - 20 wings measured per genotype). Lines are ordered by their mean wing size. For a correlation of mean wing size by genomic position of the target gene please refer to Supplementary Figure 3. Selected lines targeting genes known to affect or not affect wing morphogenesis based on previous literature are colored in orange or blue according to the color scheme in (c). While sgRNA targeting negative control genes have minimal effects on wing size, sgRNAs targeting positive control lead to a clear reduction in wing size. Phenotypes that were lethal are classified as zero and visible towards the right of the graph. (**c**) List of genes considered as positive (orange rectangle) or negative (blue rectangle) controls based on previous literature.

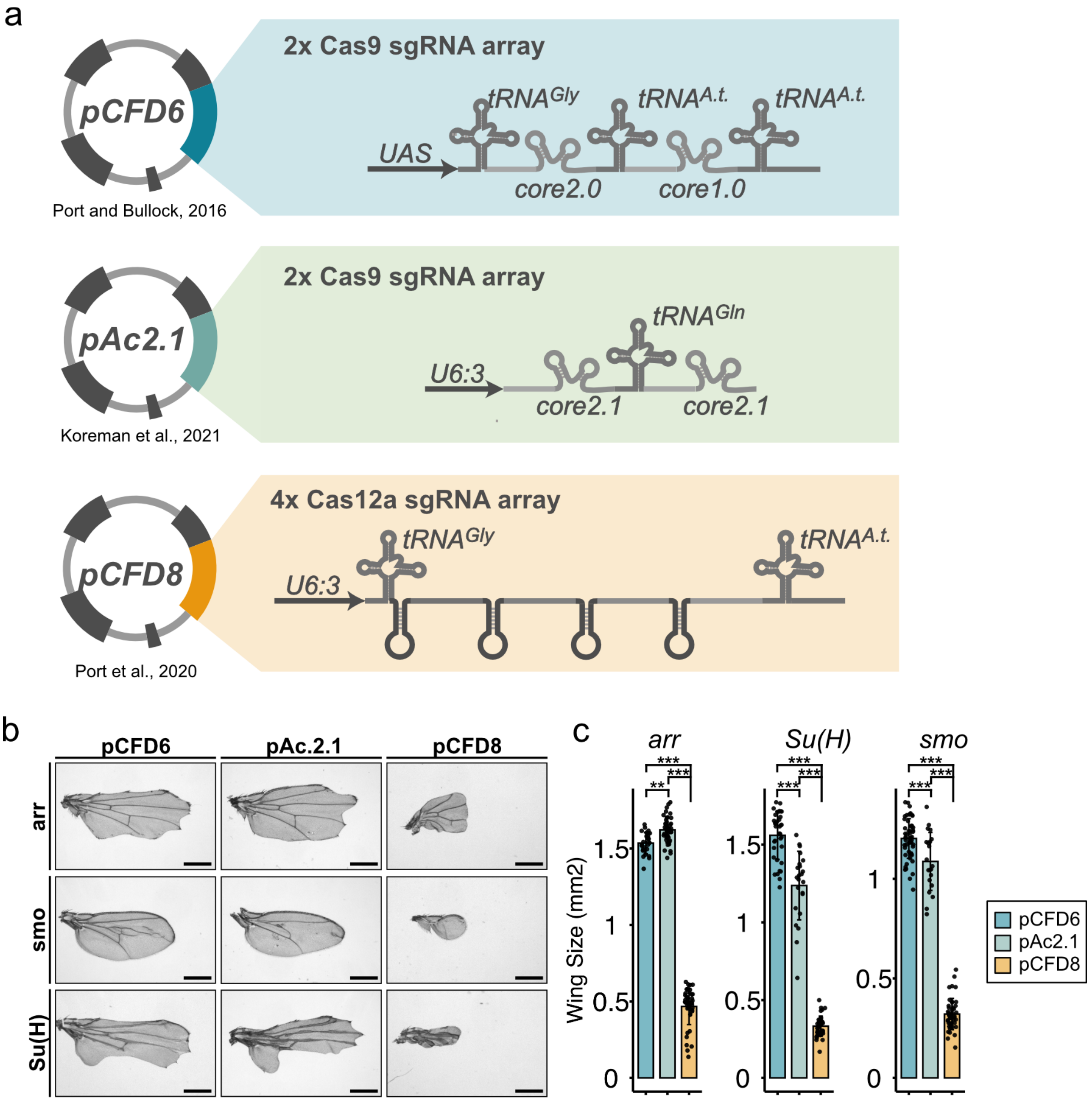

Supplementary Figure 10: Two Cas9 sgRNAs expressed from an optimized expression vector do not match knockout efficiency of HD12aCFD arrays.(**a**) Schematics of sgRNA expression vectors. *pCFD6*, used in HD_CFD and some BL sgRNA lines, expresses sgRNAs from a GAL4-inducible UAS promoter and utilizes a *Drosophila* tRNA and *Arabidopsis thaliana* tRNA for sgRNA excision. *pAc2.1* employs the constitutive *U6:3* promoter with an optimized sgRNA core sequence and *Drosophila tRNA-Gln*. The *pCFD6* constructs tested here encode two sgRNAs per gene and the same spacer sequences were cloned into *pAc2.1* for direct comparison. *pCFD8* is a Cas12a sgRNA vector featuring the *U6:3* promoter and the same tRNAs as *pCFD6*. Due to distinct sgRNA topology and PAM requirements, Cas12a does not permit spacer-matched comparisons. The *pCFD8* construct (from the HD12aCFD

library) encodes four sgRNAs. All transgenes are inserted at identical genomic landing sites. (**b**) Representative wings from animals expressing *pdm2-GAL4*, *UAS-uMCas9* or *UAS-uMCas12a*$^{+}$, and sgRNAs targeting *arr*, *Su(H)*, or *smo* from each vector. Mutagenesis with Cas12a^{+} and HD12aCFD arrays produces substantially stronger phenotypes, including wing size reduction, loss of margin tissue (*arr*, *Su(H)*), and vein defects (*smo*). Scale bars, 500 um. (**c**) Quantification of wing size from genotypes in (**b**). Data represent mean ± s.d.; individual wings are shown as dots. Expressing matched sgRNAs from *pAc2.1* yields greater size reduction than *pCFD6* for *Su(H)* and *smo* targets. However, this improved efficiency does not reach that of Cas12a^{+} with HD12aCFD arrays, which generate markedly stronger knockout phenotypes across all three genes. Note that this comparison evaluates whether existing Cas9 sgRNA resources (which typically employ one or two sgRNAs per gene) would match HD12aCFD performance if updated to use the *pAc2.1* vector, rather than directly comparing the capabilities of each vector, which would require matched sgRNA numbers. Producing quadruple Cas9 sgRNAs at scale is impractical (see Discussion). Statistical significance: **P < 0.01, ***P < 0.001 (One way ANOVA with Tukey's HSD post-hoc test).

Supplementary References:

Port, F. et al. A large-scale resource for tissue-specific CRISPR mutagenesis in Drosophila. eLife 9, e53865 (2020).

Luk, K. et al. Optimization of nuclear localization signal composition improves CRISPR-Cas12a editing rates in human primary cells. GEN. Biotechnol 1, 271–284 (2022).

Liu, P. et al. Enhanced Cas12a editing in mammalian cells and zebrafish. Nucleic Acids Res 47, 4169–4180 (2019).
