# Supplementary material for: Improved in vivo gene knockout with high specificity using multiplexed Cas12a sgRNAs
Source: Nat Commun. 2026 Jan 15;17:877. doi: 10.1038/s41467-026-68434-z (PMC12827956; doi:10.1038/s41467-026-68434-z)
Supplement: Supplementary file 2 — Description of Additional Supplementary Files [file 41467_2026_68434_MOESM2_ESM.pdf]

Description of Additional Supplementary Files

File Name: Supplementary Data 1
Description: Spreadsheet of sgRNA spacer sequences and their target genes of the HD12aCFD lines reported in this study. HD12aCFD sgRNA lines are available from the VDRC stock center (vdrc.at). Line_IDs ending with 'L' have been lost and are currently not available.

File Name: Supplementary Data 2
Description: Sequences of plasmids and primers used in this study. Plasmid sequences are listed in the first sheet and are available from the European Plasmid Repository and Addgene. Primers are listed in the second sheet of this workbook.

File Name: Supplementary Data 3
Description: Genotypes of the *Drosophila* strains used in this study.

File Name: Supplementary Data 4
Description: Code of the custom Cellpose model used to segment images of adult wings.
